# Panobinostat Effectively Increases Histone Acetylation and Alters Chromatin Accessibility Landscape in Canine Embryonic Fibroblasts but Does Not Enhance Cellular Reprogramming

**DOI:** 10.3389/fvets.2021.716570

**Published:** 2021-09-29

**Authors:** Maryam Moshref, Maria Questa, Veronica Lopez-Cervantes, Thomas K. Sears, Rachel L. Greathouse, Charles K. Crawford, Amir Kol

**Affiliations:** Department of Pathology, Microbiology, and Immunology, School of Veterinary Medicine, University of California, Davis, Davis, CA, United States

**Keywords:** canine iPSC, embryonic fibroblasts, cellular reprogramming, HDAC inhibitors, ATAC sequencing

## Abstract

Robust and reproducible protocols to efficiently reprogram adult canine cells to induced pluripotent stem cells are still elusive. Somatic cell reprogramming requires global chromatin remodeling that is finely orchestrated spatially and temporally. Histone acetylation and deacetylation are key regulators of chromatin condensation, mediated by histone acetyltransferases and histone deacetylases (HDACs), respectively. HDAC inhibitors have been used to increase histone acetylation, chromatin accessibility, and somatic cell reprogramming in human and mice cells. We hypothesized that inhibition of HDACs in canine fibroblasts would increase their reprogramming efficiency by altering the epigenomic landscape and enabling greater chromatin accessibility. We report that a combined treatment of panobinostat (LBH589) and vitamin C effectively inhibits HDAC function and increases histone acetylation in canine embryonic fibroblasts *in vitro*, with no significant cytotoxic effects. We further determined the effect of this treatment on global chromatin accessibility *via* Assay for Transposase-Accessible Chromatin using sequencing. Finally, the treatment did not induce any significant increase in cellular reprogramming efficiency. Although our data demonstrate that the unique epigenetic landscape of canine cells does not make them amenable to cellular reprogramming through the proposed treatment, it provides a rationale for a targeted, canine-specific, reprogramming approach by enhancing the expression of transcription factors such as CEBP.

## Introduction

Naturally occurring disease in companion dogs is a valuable resource for translational regenerative medicine research. Pet dogs suffer from complex and multifactorial diseases such as diabetes mellitus, cardiomyopathy, and cancer that mirror key clinical–pathological aspects of human diseases (1, 2). Like humans, dogs have a diverse genetic background within the overall population, with certain diseases being more prevalent in specific breeds that have greater genetic homogeneity (3). Moreover, pet dogs are exposed to similar environmental factors as their owners, such as a sedentary lifestyle, industrialized diets, and environmental toxins that are key in the development of spontaneous diseases (4). Finally, modern veterinary medicine offers an advanced platform to conduct translational research partnering with dog owners that are committed to providing excellent medical care to their pets and veterinary medical professionals that are highly specialized and have access to cutting-edge medical technology, mirroring the human healthcare system. As such, spontaneous diseases in dogs offer a realistic and complex translational model system.

The discovery of induced pluripotent stem cells (iPSC) in 2006 was a major breakthrough that opened new approaches to regenerative medicine by providing an unlimited source for cell therapy, drug discovery, and disease modeling (5). iPSC technology is well established in human and standard laboratory animal (e.g., mouse and rat) cells; however, the induction and stable maintenance of canine iPSCs is suboptimal and poorly understood. Although reprogramming of canine somatic cells to putative iPSCs has been described by several groups (6–10), published protocols are inconsistent and poorly reproducible. Moreover, resulting iPSCs are often poorly characterized and are likely to represent partially reprogrammed cells, as only a few of the canine iPSC publications demonstrate spontaneous *in vivo* differentiation (i.e., teratoma formation), and the contribution of canine iPSC to chimera formation has not been reported. Finally, although in our hands and others, canine embryonic fibroblasts (CEFs) are reprogrammable; adult fibroblasts remain resistant to *OCT4-SOX2-KLF4-MYC* (*OSKM*)-induced reprogramming (11, 12). We found that adult fibroblasts have a more restrictive genomic accessibility landscape compared with CEF that “locks” adult cells in their somatic fate and prevents their reprogramming and phenotypic switch (11).

In the course of reprogramming, the pioneering transcription factors *OSKM* initiate the reprogramming process, which entails marked chromatin remodeling (13). Reprogramming-associated chromatin-remodeling induces cell-fate changes through initial, middle, and maturation steps (14). Chromatin dynamic oscillates from open to closed (OC) and closed to open (CO) at different loci. To change the cell fate from somatic to a pluripotent cell, loci that are associated with somatic fate need to close (i.e., OC), whereas loci that are associated with the pluripotency fate need to open (i.e., CO). Conditions that alter the OC–CO dynamics impede reprogramming (15). Chromatin accessibility is a crucial foundation for gene expression changes during reprogramming. Histone acetylation is a common form of posttranslational modification that modulates gene expression. Acetylation results in a change in chromatin conformation, which provides a potential for the recruitment of different transcriptional factors. Mechanistically, acetylation of core histones by histone acetyltransferases weakens their interaction with DNA and results in increased chromatin accessibility, whereas deacetylation of histones by histone deacetylase (HDACs) increases the positive charges on histones and, hence, strengthens histone–DNA interaction and represses transcription (16). Histone lysine acetylation is a dynamic process balanced by the enzymatic activity of histone acetyltransferases and HDACs (16, 17). Acetylation of histone H3K9, which inhibits the methylation of the same residue, promotes H3K4 methylation. This process results in chromatin relaxation and transcriptional activation. Conversely, deacetylation of H3K9 by HDACs inhibits H3K4 methylation, which results in transcriptional repression. Also, the acetylation of histone H3K27 is an important enhancer mark that is associated with active promoters in mammalian cells (18). Hence, HDAC activity not only facilitates histone deacetylation but also impacts the overall posttranslational lysine modification (16). Various chromatin modification agents have been successfully used to enable and/or improve the reprogramming of somatic fibroblasts to iPSC in different species, including mice, pigs, and humans (19–23). We hypothesized that inhibition of histone deacetylation in canine fibroblasts enables efficient reprogramming by altering the epigenomic landscape and enabling greater chromatin accessibility. To test our hypothesis, we screened several HDAC inhibitors (HDACis) and determined their cytotoxic effect and their capacity to inhibit HDAC activity and promote histone acetylation without negatively impacting the proliferative rate. We identified that a low dose of panobinostat (LBH589) effectively inhibited HDAC activity without cytotoxicity. We determined how such a treatment impacts global chromatin accessibility *via* Assay for Transposase-Accessible Chromatin using sequencing (ATAC-seq). Our data revealed that chromatin accessibility in genomic areas associated with biological processes such as signal transduction, multicellular organism development, and DNA binding transcription factor activity was most affected. Finally, the proposed treatment did not increase the efficiency of CEF reprogramming, likely reflecting the need for a more refined approach. ATAC-seq data analysis further highlights the numerous pathways and transcription factors altered by our treatment and suggests a rationale for a targeted, canine-specific, reprogramming approach. Overall, although reprogramming efficiency was not increased, our data show that panobinostat and vitamin C treatment induces wide chromatin remodeling and that reprogramming may be enhanced by CEBP, a reprogramming enhancing transcription factor that was inhibited by our treatment and which its depletion was suggested as a barrier to canine somatic cell reprogramming (11).

## Materials and Methods

All methods were carried out in accordance with relevant guidelines and regulations. No *in vivo* experiments were included in this study.

### Cell Lines

CEF cell lines were derived from discarded embryos from terminated pregnancies upon spaying procedure at the University of California (UC), Davis, School of Veterinary Medicine exactly as previously described by our group (11). All protocols and procedures in this study were approved by the University of California, Davis, Institutional Animal Care and Use Committee. Briefly, embryonic sacs were incised with scalpels, the heads and viscera were removed, and the remaining stromal tissue was minced. The minced tissue was digested using 0.05% trypsin/ethylenediaminetetraacetic acid (Gibco) at 37°C for 45 min. The digested tissue was plated in Dulbecco's modified Eagle's medium (DMEM) (Gibco) supplemented with 20% fetal bovine serum (Corning), 0.1-mM nonessential amino acids (Gibco), 2-mM GlutaMax (Gibco), 100 U/ml penicillin, and 100 μg/ml streptomycin (Gibco). Cells were expanded and frozen in liquid nitrogen.

### Cytotoxicity Assay

CEFs (1 × 10^5^) per condition were plated in duplicate in 12-well plates. The next day, cells were treated with different concentrations of HDACis ranging from nanomolar to millimolar concentrations for 48 h. HDACis that were used in this experiment were as follows: L-ascorbic acid (Sigma), trichostatin A (Sigma), valproic acid sodium salt (Sigma), sodium butyrate (Sigma), and panobinostat (LC Laboratories). Cell number and viability were determined with a Muse Cell Analyzer (Millipore), using the Muse Count and Viability dye per manufacturer's instructions.

### MTS Assay: Colorimetric Quantification of Viable Cells

CEFs (1 × 10^5^) were plated in duplicate, in 12-well plates. The next day, cells were treated with selected concentrations of different HDACis for 72 h. To quantify cell proliferation, we used the CellTiter 96 AQ_ueous_ One Solution Cell Proliferation Assay MTS (Promega) kit per the manufacturer's instructions. Briefly, after 72 h, 400 μl of MTS solution was added to each well, plates were gently shaken to distribute the dye, and plates were incubated at 37°C for 1 h. Supernatant from each well was transferred to black clear bottom 96-well plates to record the absorbance at 490 nm using a 96-well plate reader (Molecular Device Spectramax M2e). A standard curve for each experiment was run, and the cell count was obtained by fitting the optical density measurement into the standard curve equation.

### Western Blot

Protein lysates from CEF cultures were prepared using radioimmunoprecipitation assay lysis and extraction buffer (both Thermo Scientific Pierce Protein Biology) supplemented with Halt Protease Inhibitor Cocktail (Thermo Scientific Pierce Protein Biology). Protein lysates were quantified using colorimetric assay dye (Protein Dye Concentrate, Bio-Rad) and were flash-frozen in liquid nitrogen (LN_2_) and stored at −80°C until further use. All protein samples (22 μg each) were routinely separated in a 4–12% NuPAGE Bis-Tris mini-gel (Invitrogen) using MOPS Running Buffer (Invitrogen) at 200 V for 2 h and then transferred to a polyvinylidene difluoride membrane at 30 V for 2 h. Membranes were blocked for 1 h with One Block Western-CL Blocking Buffer (Genesee Scientific) and then probed overnight at 4°C, with the primary antibody in 5% bovine serum albumin in Tris-buffered saline buffer. The following day, blots were washed with Tris-buffered saline with 0.1% Tween-20 detergent buffer and incubated in secondary antibodies in the same blocking buffer for 1 h. For Histon H3, blots were stripped with stripping buffer (Thermo Fisher) for 10 min at room temperature and blocked for 1 h followed by primary antibody incubation as mentioned earlier. Primary (24–27) and secondary (28) antibodies used are listed in Table 1. Blots were visualized using a ProteinSimple Fluorchem E Imager. Immunoblots all experienced the same transfer conditions, primary/secondary antibody concentrations, and exposure times.

**Table 1:** Antibodies used for western blots.

**Target**	**Manufacturer**	**Catalog no**.	**Type**	**Concentration**
**Primary antibodies**				
β-tubulin	Cell signaling technology	2,146	Rabbit Monoclonal	1:1,000
Histone H3	Cell signaling technology	9,715	Rabbit Monoclonal	1:1,000
Lamin B1	Cell signaling technology	13,435	Rabbit Monoclonal	1:1,000
Acetyl-Histone H3 (Lys27)	Cell signaling technology	9,927	Rabbit Monoclonal	1:1,000
Acetyl-Histone H3 (Lys9)	Cell signaling technology	9,927	Rabbit Monoclonal	1:1,000
**Secondary antibodies**				
Anti-rabbit IgG, HRP-linked antibody	Cell signaling technology	7,074		1:5,000

### Lentivirus Production

OCT4-KLF4-SOX2-IRES-MYC (OKSIM) (29) plasmid, a gift from José Cibelli (Addgene plasmid # 24603), was used for lentivirus production. Plasmid identity was confirmed by restriction enzyme digestion, and lentivirus packaging was performed by the UC Davis Vector Core Facility (Stem Cell Program, UC Davis Medical Center) using helper plasmids Tat/Rev/Gag-Pol (psPAX2), VSV-G (pMD2.G), and Lonza Ultraculture Packaging Media.

### Histone Deacetylase Activity Assay

CEFs were treated with HDACi compounds for 48 h, as described earlier. Nuclei were isolated and extracted from 1 × 10^7^ cells according to the manufacturer's instructions (Histone Deacetylase Activity kit, Abcam, ab156064) (30). Briefly, cells were resuspended in 1 ml of lysis buffer followed by centrifugation over a 30% sucrose solution. Nuclei pellets were washed and resuspended in an extraction buffer. Samples were sonicated for 30 s (EpiShear Q120AM probe sonicator, Active Motif) followed by a 30-min incubation on ice. The supernatants (i.e., crude nuclear extracts) were flash-frozen in LN_2_ and stored at −80°C until further use. Small aliquots of the supernatants were used to determine protein concentration by the Bradford method using protein dye concentrate. All experiments were carried out according to the manufacturer's instructions, following the two-step method and using the controls provided by the kit. The fluorescence intensity was measured in a microplate fluorescence reader (Molecular Devices FilterMax F3) at Excitation/Emission = 350–380/440–460 nm.

### Reprogramming of Canine Embryonic Fibroblast

CEF cells (0.6 × 10^5^) (passage ≤ 2) were plated in six-well plates on day−1 postinfection (pi). Cells were transduced on day 0 with lentivirus (OKSIM), at a multiplicity of infection = 80 with 10 μg/ml Polybrene (Millipore) in complete DMEM (Gibco) media. Media was replaced everyday pi with DMEM/F12 (Gibco) supplemented with 20% KnockOut Serum Replacement (Gibco), 0.1-mM nonessential amino acid, 2-mM GlutaMax, 100 U/ml penicillin, and 100 μg/ml streptomycin (Pen/Strep), 0.075-mM β-mercaptoethanol (Sigma-Aldrich), 8 ng/ml bFGF, and 10 ng/ml of LIF (Peprotech) with or without treatment (panobinostat 1 nM and vitamin C 150 μM). Upon confluency, between day 6 and 7 pi, cells were dissociated with TRypLE Express (Gibco), and 2 × 10^5^ cells were plated on each 10 -m tissue culture plate with a feeder layer of 1 × 10^6^ fresh irradiated mouse embryonic fibroblast (iMEF) and the media replaced. Colonies were counted between days 14 and 21 pi.

### Assay for Transposase-Accessible Chromatin Using Sequencing Library Preparation

CEF cells were plated in 10-cm plates overnight. One day after, the media was replaced with DMEM complete medium with or without treatment for 48 h. DNA libraries were prepared exactly as previously described (11). Briefly, 5 × 10^4^ cells were lysed in cold lysis buffer, and the isolated nuclei pellets were resuspended in the transposase reaction mix (Illumina) and incubated for 60 min at 37°C, with agitation at 300 rpm. Samples were purified using a MinElute column (Qiagen), and the libraries were polymerase chain reaction-amplified using SsoFast EvaGreen Supermix (Bio-Rad Laboratories) and Nextera polymerase chain reaction primers. We determined optimal library amplification exactly as previously described (11). All libraries were amplified for a total of 15–21 cycles, as described previously (11, 31, 32). We used Agencourt AMPure XP beads (Beckman Coulter) to clean the libraries. Finally, libraries were quantified in a BioAnalyzer 2100 (Agilent Technologies) and sequenced in an Illumina HiSeq4000 system in a paired-end 150-bp run. All the experiments were carried out two times and with three different CEF cell lines.

### Assay for Transposase-Accessible Chromatin Using Sequencing Data Analysis

The data preprocessing step, including fragment cleaning, duplicate removal, adapter trimming, and pair-ended reads overlapping, was done using HTStream (33). We kept only unique reads mapping to a single genomic location and strand in the CanFam3.1 canine genome assembly using Burrows–Wheeler Alignment tools for alignment, SAMtools (34) for filtering, and macs2 (35) for peak calling, as previously described (11). Quality control was performed with HTStream and MultiQ (36). We used deeptools (37) for creating BigWig files and npz matrix files for the construction of principal component analysis plots as previously described (11).

After alignment, the DiffBind (version 2.12.0) (38) package in R was used, along with edgeR (version 3.26.8) (39) to call differential binding sites (between treatment and control) while taking the replicates into consideration. GREAT (version 4.0.4) (40) was used to find the regulatory domains for each site and then BEDTools (version 2.29.2) (41) to intersect those domains with annotated genes. For differential openness analysis, the binding sites were split into three categories for each comparison: Sites with a positive log-fold change having a false discovery rate (FDR) adjusted *p* < 0.05, sites with a negative log-fold change having an FDR adjusted *p* < 0.05, and sites with an FDR adjusted *p* ≥ 0.05.

We performed Pearson correlation distance calculation of the differential openness data with Cluster 3.0 (42) and used Java TreeView (43) for visualization to create the hierarchical clustering graph as we previously described (11). Gene ontology (GO) enrichment analyses of differential openness results were conducted using Kolmogorov–Smirnov tests, as implemented in the Bioconductor package topGO (44). Analyses were conducted for the biological process, molecular function (MF), and cellular component GO ontologies. We graphed the first 15 GO terms with *p* < 0.05 from each of these major ontologies to represent a view of the top GO terms enriched in our dataset. Next, we used PANTHER (45) for GO term reclassification of the genes annotated within the following GO terms of interest: “Wnt-signaling pathway, “stem cell development,” “histone H4 acetylation,” “regulation of cell differentiation,” “cell population proliferation,” “regulation of telomere maintenance,” “regulation of somatic stem cell population maintenance,” “cell differentiation,” “histone binding,” and “signaling receptor binding.” Genes with adjusted *p* < 0.05 were reclassified within the “Pathway/TGF-β,” “Pathway/Wnt,” “Pathway/PDGF,” and “MF/transcription regulator activity” GO classifications.

Transcription factor (TF) motif enrichment was examined in the peaks that reached statistical significance (adjusted *p* < 0.05) for the differential openness analysis. Genomic sequence was extracted for each site, per category, using BEDTools. The extracted genomic sequence from the sites with a *p* ≥ 0.05 was used as background data in an enrichment analysis performed with HOMER (34) (version 4.10), running findMotifs.pl with FASTA files (-fasta). We kept the first 25 enriched motifs found with both *p* < 0.0001 and fold enrichment (FE = % target/% background) over 1.2. We used GraphPad Prism 8 and Microsoft Powerpoint version 16.42 for figure presentation.

### Alkaline Phosphatase Staining

Alkaline phosphatase (AP) activity was assessed using the Alkaline Phosphatase Staining Kit II (StemGent) (46), according to the manufacturer's protocol. Images were captured under a light microscope (Olympus) using ToupView software.

### Statistical Analyses

Data were evaluated for normal distribution using commercially available software (GraphPad Prism 8). For data sets that followed a normal distribution pattern, a one-way analysis of variance with the Geisser–Greenhouse correction was used. A nonparametric method, the Kruskal–Wallis test, was used for data sets that did not follow a normal distribution pattern. The two-stage step-up method of Benjamini, Krieger, and Yekutieli (47) for controlling the FDR was used for multiple comparison corrections across all the data sets. For all statistical tests, the FDR adjusted *p* of <0.05 was considered statistically significant.

## Results

### Identification of Noncytotoxic Concentration Ranges of Histone Deacetylase Inhibitors Compounds in Canine Fibroblasts

**Figure 1:**
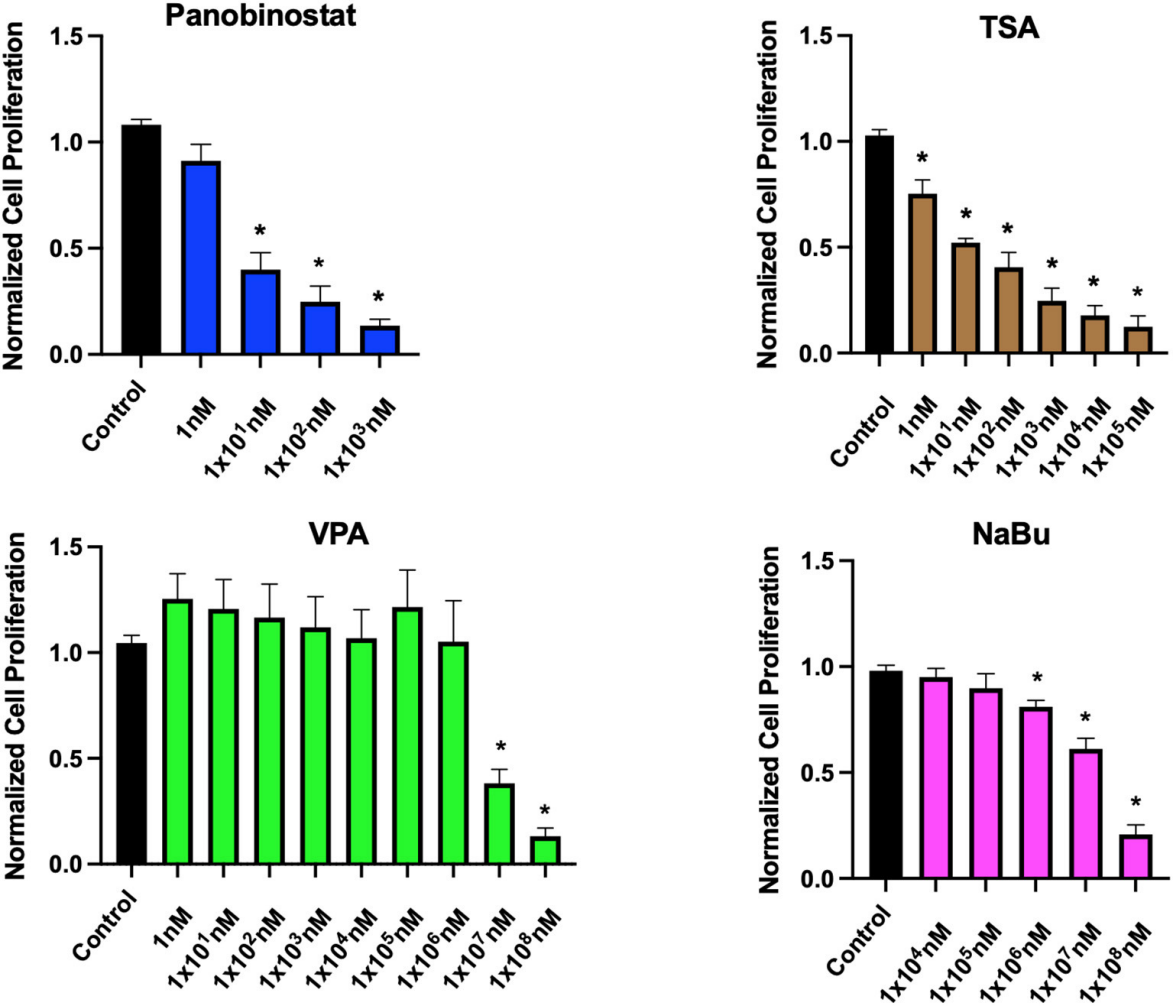
Identification of noncytotoxic concentration ranges of HDACi compounds in CEF. CEFs were treated with a wide range of HDACi concentrations for 48 h. Cells were counted, and data were normalized to non-treated control. Each experiment was conducted six times using three different CEF lines. Data are presented as mean ± SEM. Two-stage step-up method of Benjamini, Krieger, and Yekutieli for controlling false discovery rate was used for multiple comparison corrections. *False discovery rate adjusted *p* < 0.05.

**Figure 2:**
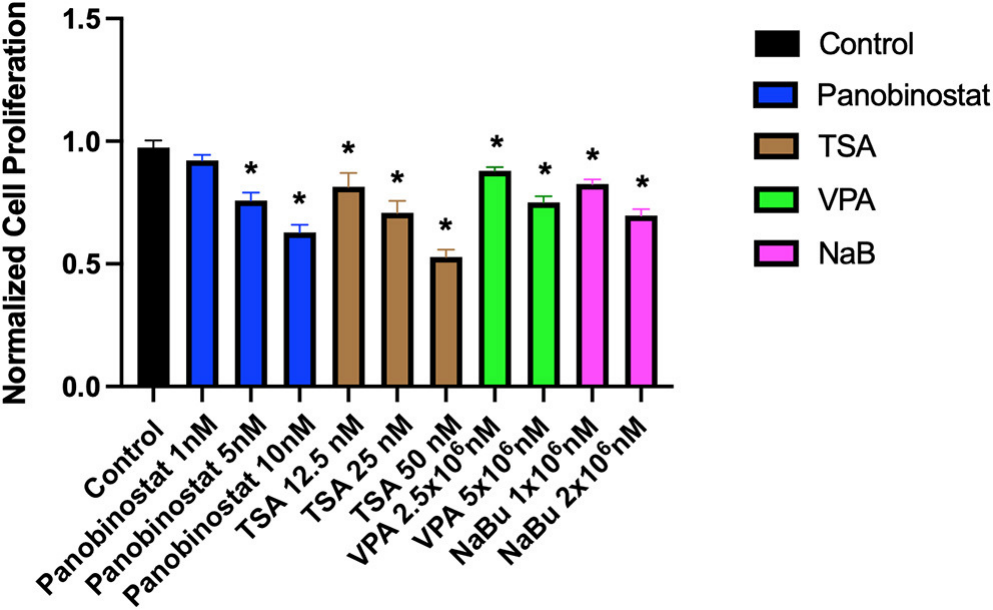
Identification of a narrow range of HDACi concentration that does not impact CEF proliferative rate. CEFs were treated with a narrow range of HDACi concentrations for 72 h. Each experiment was conducted six times using three different CEF lines. Data were normalized to non-treated control. Data are presented as mean ± SEM. Two-stage step-up method of Benjamini, Krieger, and Yekutieli for controlling false discovery rate was used for multiple comparison corrections. *False discovery rate adjusted *p* < 0.05.

To determine optimal HDAC inhibition, we first tested a wide range of HDACi compounds and concentrations to identify and narrow the range of concentrations in which the tested compounds do not have a detrimental cytotoxic effect (Figure 1). Concentrations higher than 1 × 10^1^-nM panobinostat, 1 × 10^6^-nM valproic acid sodium salt (VPA), and 1 × 10^5^-nM sodium butyrate induced significant cytotoxicity. Trichostatin A (TSA) was cytotoxic at all concentrations (Figure 1). We then followed up with additional proliferation assays to define the cytotoxic threshold more precisely (Figure 2). We found that 1-nM panobinostat did not inhibit cell proliferation significantly and was considered for further analysis.

### Panobinostat Effectively Inhibits Endogenous Histone Deacetylase and Promotes Chromatin Acetylation

**Figure 3:**
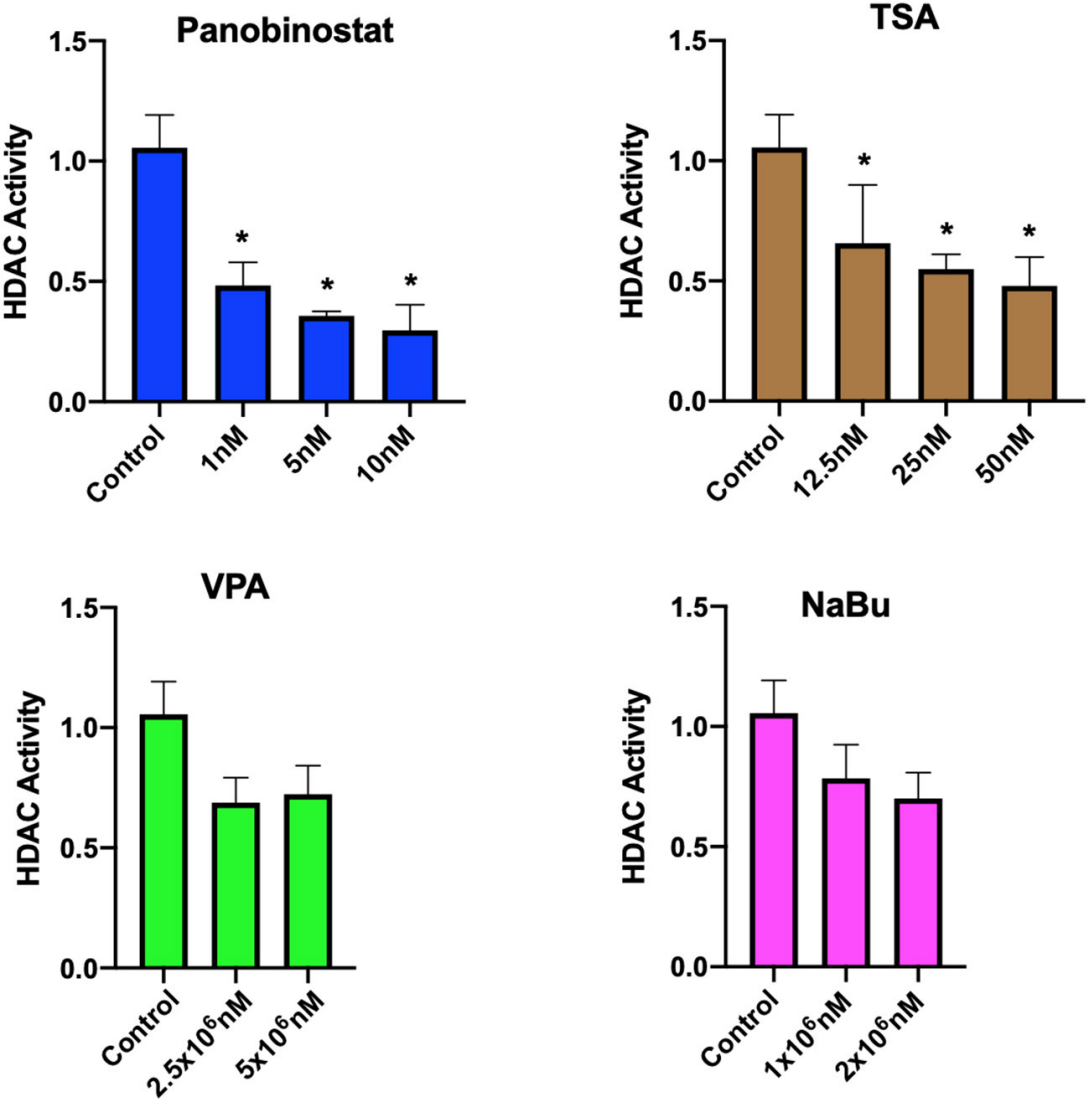
Assessment of HDAC activity in CEF upon HDACi treatment. HDAC activity of crude nuclear extract from 1 × 10^7^ cells was determined in untreated cells and HDACi treated CEF. Data are normalized to untreated control. Each experiment was conducted four times using two different CEF lines. Data are presented as mean ± SEM. Two-stage step-up method of Benjamini, Krieger, and Yekutieli for controlling false discovery rate was used for multiple comparison corrections. *False discovery rate adjusted *p* < 0.05.

**Figure 4:**
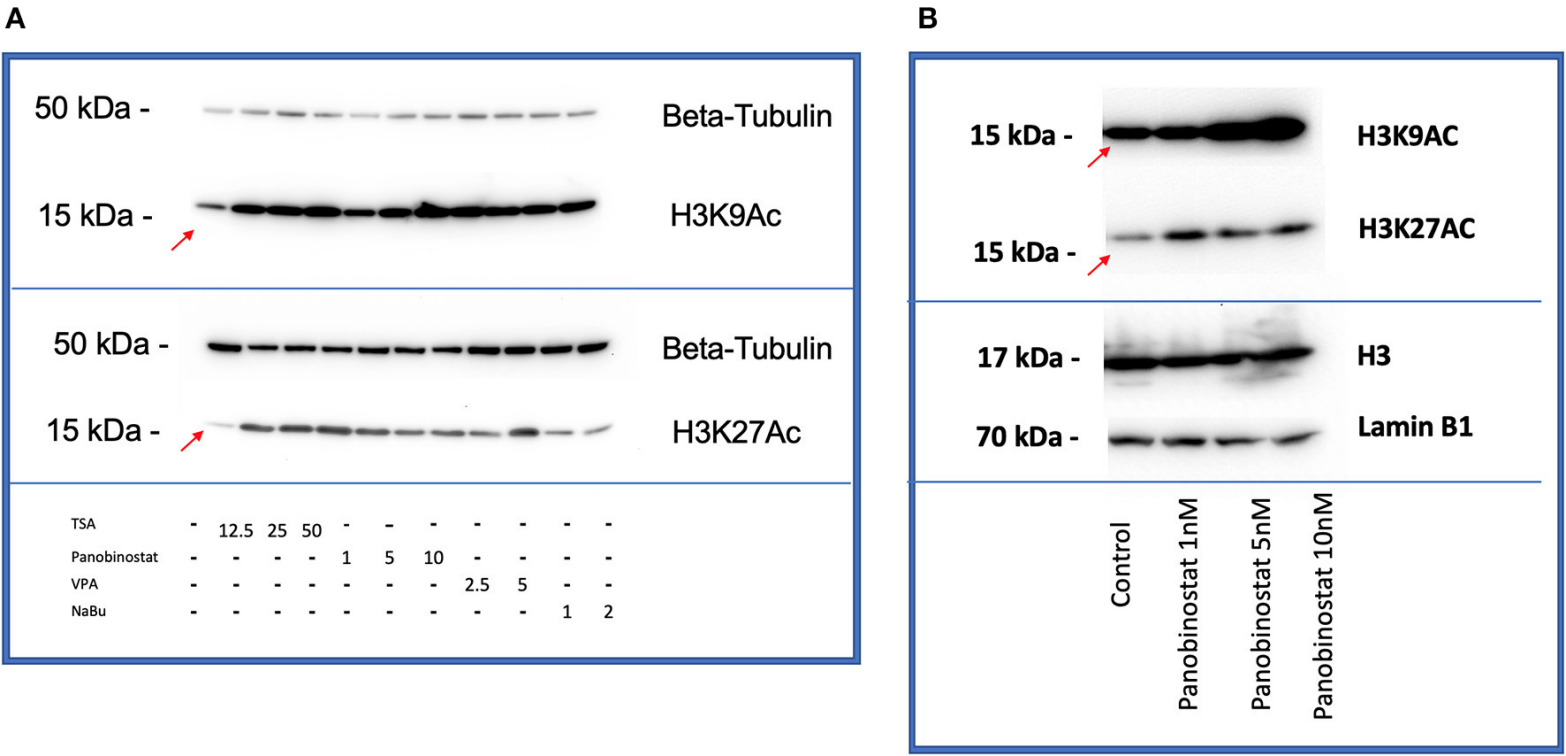
HDACi effectively increases H3K9 and H3K27 histone acetylation in CEF. **(A)** All compounds showed an increase in acetylation of H3K9 (top) and H3K27(bottom) by Western blot comparing with non-treated control band (red arrows are pointing at bands related to non-treated controls). β-tubulin was used as a loading control. Blots were repeated two times using two different CEF lines. **(B)** Panobinostat 1-, 5-, and 10-nM treatment increased H3K9, H3K27 acetylation compared with non-treated control (red arrows are pointing at bands related to non-treated controls). Total H3 and Lamin B1 were used as loading controls.

After determining CEF-specific cytotoxic concentrations, we aimed to determine if these reported HDACi compounds have an effective HDAC inhibitory effect in CEF. Our data show that all the tested panobinostat concentrations (1, 5, and 10 nM), as well as TSA concentrations (12.5, 25, and 50 nM), significantly reduced HDAC activity, whereas VPA and sodium butyrate did not inhibit endogenous HDAC in the tested concentrations (Figure 3). To compare acetylated histone abundance between samples, we chose to determine H3K9 and H3K27 acetylation due to their relevance to reprogramming. Acetylated H3K27 (H3K27ac) distinguishes active from inactive enhancers (48). Also, acetylation of H3K9 (H3K9ac) in promoter areas has been broadly reported to facilitate gene transcription (49). Our data illustrate that histone acetylation was increased in CEF treated with TSA and panobinostat in all tested concentrations (Figure 4). Altogether, although TSA effectively inhibited endogenous HDAC and promoted histone acetylation, only panobinostat treatment (1 nM) did not blunt cell proliferation while effectively inhibiting HDAC activity and promoting histone acetylation.

As a high proliferative rate facilitates cellular reprogramming, we further investigated if the combination of vitamin C (ascorbic acid) and panobinostat 1 nM promotes canine somatic cell reprogramming. Vitamin C improves the speed and efficiency of both mouse and human somatic cell reprogramming by alleviating senescence and affecting the epigenetic landscape of the adult cells. Specifically, it enhances the catalytic activity of Jumonji-C domain-containing histone demethylases and ten-eleven translocations and therefore promotes histone and DNA demethylation in somatic cells. These events allow pluripotency genes to be turned on while simultaneously erasing the epigenetic memory of the adult cell state (50–52). We first tested a wide range of concentrations of vitamin C, ranging from 1 nM to 100 mM, and chose a range that was not only nontoxic but also increased cell proliferation in CEF compared with the non-treated control (Supplementary Figure 1). We further aimed to determine if combining vitamin C with 1-nM panobinostat could still promote cell proliferation, a key feature that is supportive of effective reprogramming. We determined that the combination of vitamin C and 1-nM panobinostat did not decrease the proliferation rate. We hypothesized that treatment of primary canine fibroblasts with HDACis increases global histone acetylation and, subsequently, reprogramming efficiency. Therefore, we investigated if adding vitamin C to the media impacts the HDAC activity and histone acetylation alone and, when combined with panobinostat, in the tested concentrations. Adding the vitamin C to the media in the presence of 1-nM panobinostat did not affect the HDAC activity and the histone acetylation compared with 1-nM panobinostat without vitamin C (Supplementary Figure 2).

### Assay for Transposase-Accessible Chromatin Using Sequencing Analysis Identifies Consistent and Reproducible Chromatin Accessibility Remodeling Upon Treatment of Canine Embryonic Fibroblast With Panobinostat and Vitamin C

We hypothesized that HDAC inhibition and augmented chromatin acetylation would lax chromatin structure and enable greater chromatin accessibility. To test our hypothesis, we studied the changes in the chromatin accessibility landscape of CEF upon treatment *via* ATAC-seq. We sequenced three different CEF cell lines in duplicates, treated vs. non-treated.

**Figure 5:**
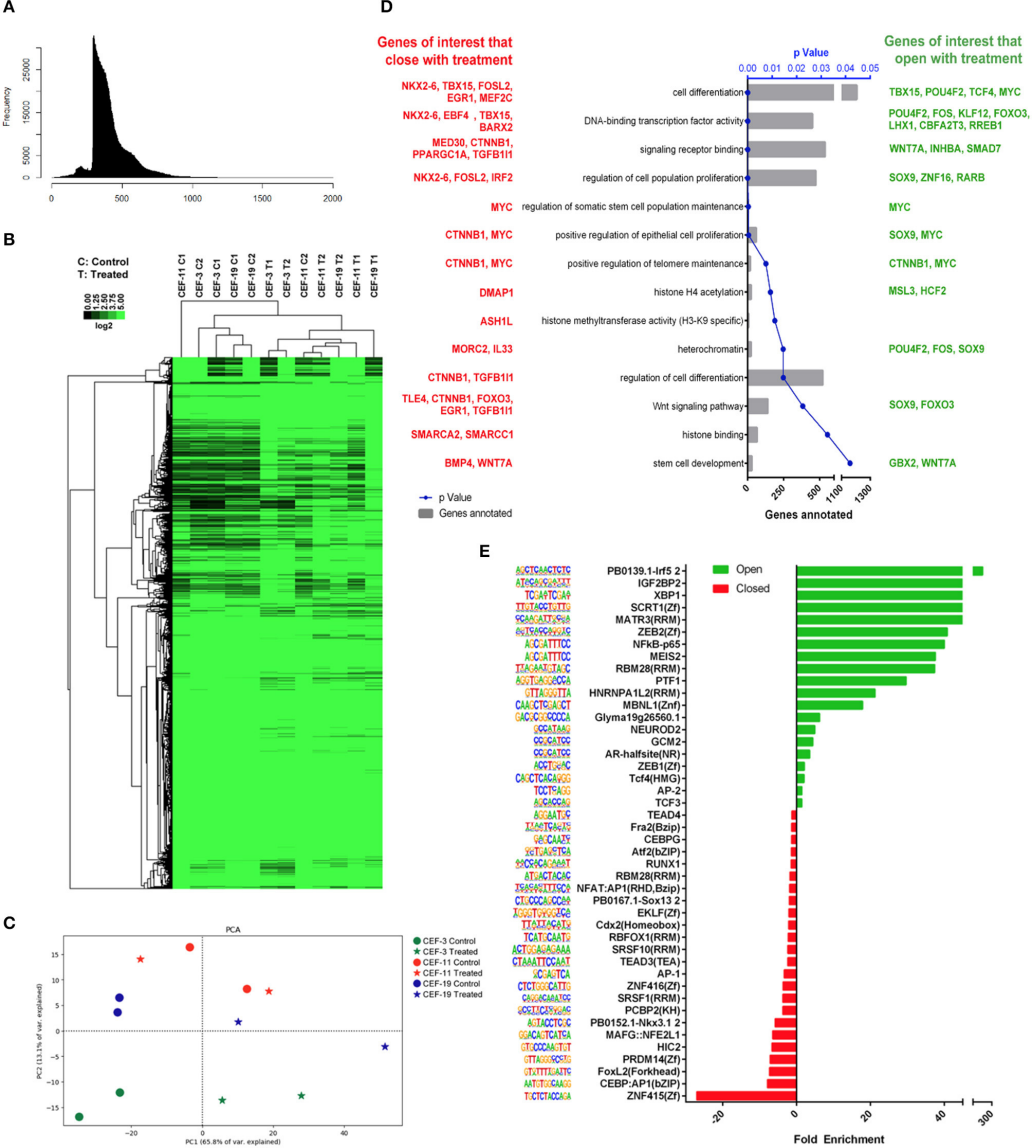
Remodeling chromatin accessibility upon treatment of CEF with panobinostat and vitamin C. **(A)** Nucleosomal pattern of sequenced library. **(B)** Unsupervised hierarchical clustering and heatmap comparisons of ATAC-seq peaks from CEF *vs*. treated CEF. **(C)** Principal component analysis plot showing that direction of data changed (two replicates, three CEF cell lines) upon treatment. **(D)** GO terms of interest reclassification by PANTHER. Genes with adjusted *p* < 0.05 reclassified within “Pathway/TGF-β,” “Pathway/Wnt,” “Pathway/PDGF,” and “MF/transcription regulator activity” GO classifications. Red: Genes of interest that became closed upon treatment. Green: Genes of interest that became more open upon treatment. **(E)** TF motifs enrichment upon treatment.

The nucleosomal pattern of the sequenced ATAC-seq library showed periodical peaks representing the expected enrichment of mono-, di-, and tri-nucleosomes (~200, 400, and 600 bp, respectively, Figure 5A). Unsupervised hierarchical clustering and heatmap comparisons of the ATAC-seq results from control CEF *vs*. treated CEF suggested that consistent chromatin accessibility changes occurred in response to treatment (Figure 5B). The principal component analysis plot illustrated that treated samples are mostly mapped on the right side of the cartesian space and display a consistent distance between non-treated controls and treated samples (Figure 5C). GO term analysis demonstrated that the most significantly overrepresented GO terms in treated samples were related to cell differentiation, multicellular organism development, regulation of transcription factors, DNA binding, and DNA binding transcription factor activity (Supplementary Figure 3). We investigated if the treatment group had a significantly more accessible chromatin structure relative to the control group across regulatory regions as previously described (40). We reclassified the GO terms and reported a list of genes associated with peaks with a significant adjusted *p* ≤ 0.05. Of specific importance was the transforming growth factor (TGF)-β superfamily secreted ligands TGFB1 and BMP4 that were significantly more closed in the treatment group (Figure 5D).

TF binding motif analysis showed that several reprogramming and cell identity-associated TF binding motifs, including TEAD4, TEAD3, AP1, NFAT: AP1, AP2, CEBP, MEIS2, and NF-κB, were enriched upon treatment (Figure 5E). Overall, our data demonstrate that vitamin C and panobinostat treatment changed the chromatin accessibility landscape of CEF consistently. Moreover, it appears that our treatment uniquely targeted GO terms and transcription factor binding motifs that may be critical for cellular reprogramming, such as differentiation, development, and regulation of transcription factors.

### Vitamin C and Panobinostat Treatment Did Not Change the Reprogramming Efficiency of Canine Embryonic Fibroblast

**Figure 6:**
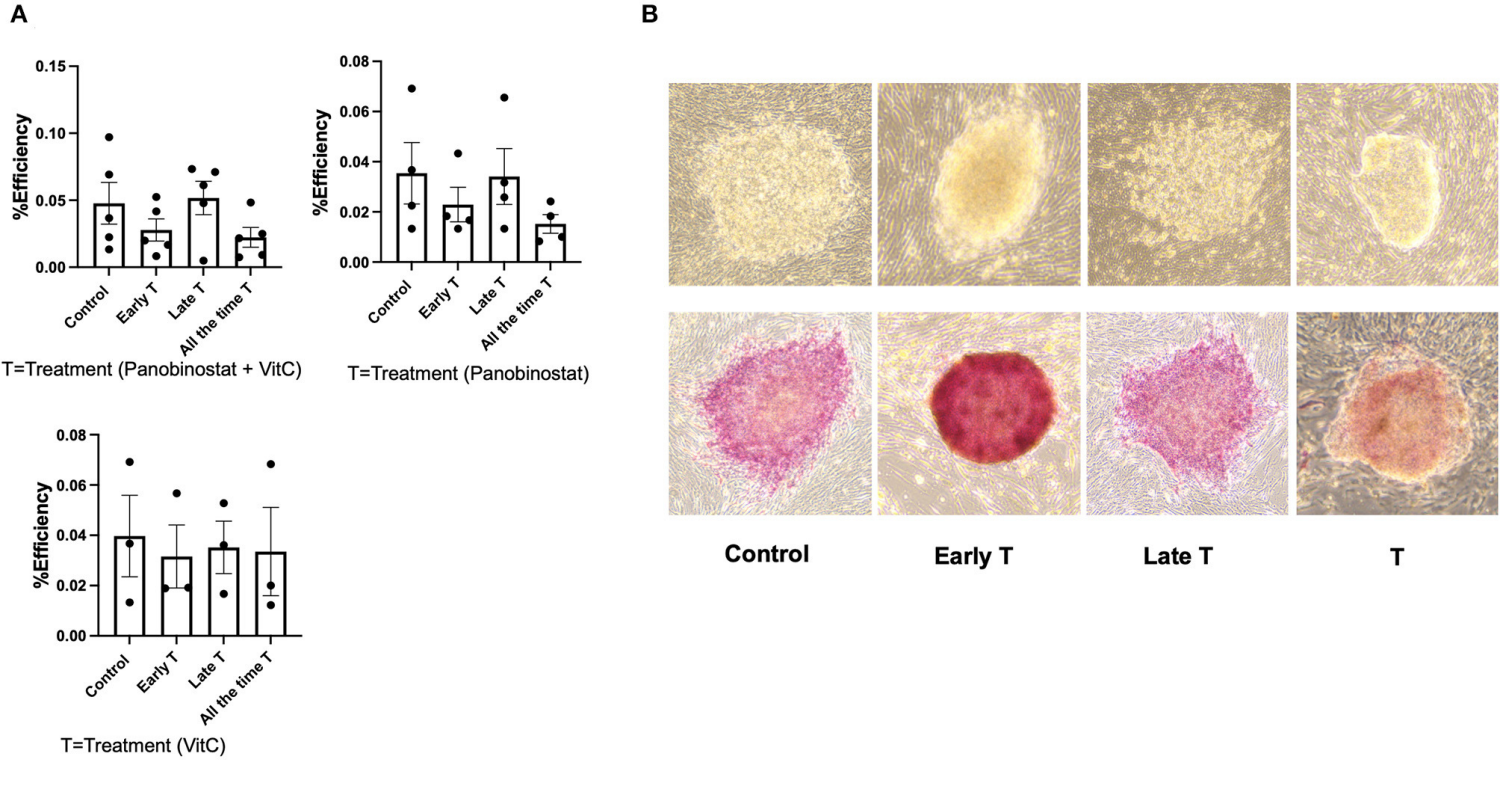
Panobinostat and vitamin C treatment did not change reprogramming efficiency of CEF. **(A)** Reprogramming efficiency as determined by number of primary iPSC colonies formed, normalized to number of CEF plated. Data are presented as mean ± SEM. Experiment was conducted five times using two different CEF cell lines for panobinostat 1 nM and vitamin C treatment group. Experiments were conducted three times for groups treated with panobinostat 1 nM only and vitamin C only. We had three different treatment groups based on time that cells received treatment as follows: 1— “All the time T” group was treated with vitamin C (150 μM) and panobinostat (1 nM) from day 1 pi and until end of experiment when primary colonies were counted (14–15 days pi). 2— “Early T” group was treated with same compounds and concentrations from day 1 to 6 and 7 pi, until cells were passed on to an irradiated mouse embryonic fibroblast (iMEF) feeder layer. 3— “Late T” group was treated with same compounds and concentrations, but treatment began after cells was passed on to iMEF and until time point in which primary colonies were counted (14–15 days pi). **(B)** Top row: Morphology of ciPSC colonies comparing treatment at different time points. Bottom row: alkaline phosphatase staining of ciPSC colonies treated at different timepoints.

We had previously reported on the OSKM-mediated reprogramming of CEF to putative canine iPSC using a lentiviral vector (11). Given our findings, we wanted to determine if panobinostat and vitamin C treatment can enhance OSKM-mediated reprogramming efficiency in CEF. We treated the lentivirus-infected CEF at three different time points. The “Treatment” group was treated with vitamin C (150 μM) and panobinostat (1 nM), vitamin C (150 μM) only, or panobinostat (1 nM) only from day 1 pi and until the end of the experiment when primary colonies were counted (14 to 15 days pi). The “Early treatment group” was treated with the same compounds and concentrations from day 1 to day 6 or 7 pi until cells were passed on to an iMEF feeder layer. The “Late treatment” group was treated with the same compounds and concentrations, but treatment began after the cells were passed on to iMEF and until the time point in which primary colonies were counted (14 to 15 days pi). We did not see any significant difference in reprogramming efficiency in any of our treatment protocols (Figure 6). Our attempts to use panobinostat and vitamin C treatment to facilitate OSKM-mediated reprogramming of adult canine fibroblasts were further unsuccessful (data not shown).

## Discussion

Canine-specific somatic cell reprogramming regulators are poorly understood, hindering the generation of robust and reproducible canine somatic cell reprogramming protocols. Very recently, two groups described the generation of canine iPSC and have demonstrated teratoma formation (10, 53). Interestingly, although both groups used very different reprogramming protocols, both have used forskolin, a cAMP activator, in their reprogramming media. Changing the chromatin landscape with small molecule inhibitors of HDACs, DNA methyltransferases, and histone methyltransferases all increased reprogramming efficiencies in somatic cells of mice and humans (21, 51, 54). Huangfu et al. reprogrammed primary human fibroblasts using VPA, class I HDACi (55), and retroviral constructs harboring the pluripotency TF OCT4, SOX2, and KLF4. They reported that VPA treatment not only increased the number of iPSC colonies by 50-fold but also allowed efficient induction of iPSC cells without the need to deliver c-MYC, a fundamental oncogene and member of the classic OSKM reprogramming TFs.

Panobinostat is a pan-HDACi (55), specifically against classes I, II, and IV, with low IC_50._ Panobinostat has been investigated as an anticancer therapy for hematologic and solid tumors in preclinical models and clinical use (55, 56). Dias et al. investigated the cytotoxic effect of panobinostat in different canine lymphoma cell lines (57). Consistent with our data, they showed that both panobinostat and TSA exhibit antiproliferative and cytotoxic activity at concentrations lower than their IC_50_, 5 and 67 nM, respectively (57). They also confirmed that panobinostat treatment increases histone acetylation *in vitro* (57). Here, we show that panobinostat treatment increased histone acetylation on lysine 9 and 27. Within this framework, we aimed to find a concentration of panobinostat that effectively inhibits the HDAC activity without compromising cellular proliferation. Our data confirm that the 1-nM concentration of panobinostat is the optimal concentration to achieve our aim. To improve the effectiveness of the treatment, we supplemented the media with 150-μM vitamin C as many of the chemically defined media contain vitamin C for its antioxidant properties, which supports cell growth. Esteban et al. reported that adding vitamin C to the culture medium alleviates cellular senescence during reprogramming and, when combined with VPA, generated more iPSC colonies. They concluded that vitamin C enhanced the reprogramming of somatic cells to iPSCs in both mice and humans (58). To the best of our knowledge, vitamin C does not affect histone acetylation directly. To make sure adding vitamin C does not negatively affect panobinostat's HDACi activity, we tested the proliferation rate, HDAC activity, and histone acetylation when vitamin C was combined with panobinostat in the tested concentrations (Supplementary Figure 2). We noticed that adding vitamin C to our media helped the growth of canine primary fibroblast. Because cell proliferation rate is important for reprogramming, we kept the vitamin C in the composition of the media. Consistent with the notion that chromatin accessibility often coincides with an increase in gene expression in eukaryotes (59, 60) and following the mouse and human somatic cell trajectory toward reprogramming (15, 61, 62), we hypothesized that HDACi treatment alters the global chromatin landscape, increasing its accessibility and flexibility.

TF binding motif enrichment analysis of our data revealed that upon the treatment, there was an enrichment of several TF binding motifs, which play important roles in the process of reprogramming. Some TF binding motifs that were enriched in CEF after treatment, such as TEAD4 (63), TEAD3 (15), AP1 (15, 64), NFAT: AP1 (65), AP2 (66), CEBP (67, 68), MEIS2 (69), and NF-κB (70, 71) are involved in the establishment of pluripotency. Specifically, the AP-1 family has previously been reported to impede somatic cell reprogramming (15). Our data indicate that panobinostat and vitamin C treatment closed the AP-1 family binding motifs and opened AP-2 binding motifs. Pastor et al. reported that AP-2 TF binding motifs are enriched at the naïve pluripotent stage (72). They further reported that the OCT4 enhancer region was enriched for one of the AP-2 family member (TFAP2C) binding motifs, indicating that TFAP2C not only established and maintained naïve pluripotency in human cells but also regulated OCT4 expression (72).

Moreover, Di Stefano et al. described that CEBP co-expression with OSKM increased the reprogramming efficiency of mouse B cells to iPSCs by 11-fold (68). Our data demonstrate that CEBP binding motifs were closed by treatment, suggesting a decrease in the functional capacity of this critical TF. Previous work from our lab indicated that accessible CBEP binding motifs are enriched in CEF and canine iPSC compared with adult fibroblasts, suggesting that enhancing CBEP activity may promote canine somatic cell reprogramming (11).

Recently, Xing et al. demonstrated the role of TEAD4 as an effector of reprogramming. Depletion of TEAD4 decreased reprogramming efficiency and established the pivotal role of TEAD4 at the intermediate and late stages of reprogramming (63). Our data indicate that panobinostat and vitamin C treatment closed TEAD4 binding motifs. Overexpression of TEAD4 could potentially enable more efficient reprogramming of the CEF.

TF-induced reprogramming has to achieve two key tasks, namely the extinction of the somatic program, which is maintained by counteracting TFs, and the induction of a stable pluripotent state. Our study illustrates that panobinostat and vitamin C treatment of CEF contributes to the openness and closeness of related TF binding motifs that are crucial in pluripotency. As such, our data create a roadmap proposing potential candidate genes for inhibition or activation to improve the reproducibility of canine iPSC reprogramming.

Finally, treatment of CEF with HDACi did not improve CEF reprogramming efficiency, indicating that global HDAC inhibition does not promote somatic cell reprogramming in the dog and that a more targeted approach is required. Consistent with our findings, Kim et al. showed that treating canine oocytes with suberoylanilide hydroxamic acid, an HDACi, in an effort to increase somatic cell nuclear transfer efficacy in the context of cloning, increased the acetylation of H3K9 but did not improve cloning efficiency (73). These collective observations may suggest that although histone acetylation can be readily enhanced in canine cells, other factors (i.e., such as histone/DNA methylation) or activation of specific genomic targets are likely to be required for canine somatic cell reprogramming.

Overall, these findings indicate that the exposure of CEF to panobinostat and vitamin C alters the expression of DNA-binding, chromatin-binding, and signaling factors, many of which are associated with the acquisition of pluripotency. Interestingly, this alteration in chromatin accessibility landscape did not increase the reprogramming efficiency, exemplifying the unique canine epigenomic landscape. Our data further highlight candidate pathways and specific TFs that function as reprogramming barriers, which could be targeted to enhance canine-specific somatic cell reprogramming in future studies.

## Data Availability Statement

The datasets presented in this study can be found in online repositories. The names of the repository/repositories and accession number(s) can be found below: NCBI [accession: PRJNA742074].

## Ethics Statement

The animal study was reviewed and approved by UCD Institutional Animal Care and Use Committee (IACUC).

## Author Contributions

MM and AK conceived and designed the research. MM performed the experiments. MQ, VL-C, TKS, RLG, and CKC assisted with the experiments. MM and MQ analyzed the data. MQ wrote the ATAC-seq data analysis method. MM and AK wrote the manuscript. All authors reviewed the manuscript.

## Funding

MM was supported by the Graduate Student Support Program UC Davis-School of Veterinary Medicine. AK was supported by UC Davis, Center for Companion Animal Health grants 2019-25-F.

## Conflict of Interest

The authors declare that the research was conducted in the absence of any commercial or financial relationships that could be construed as a potential conflict of interest.

## Publisher's Note

All claims expressed in this article are solely those of the authors and do not necessarily represent those of their affiliated organizations, or those of the publisher, the editors and the reviewers. Any product that may be evaluated in this article, or claim that may be made by its manufacturer, is not guaranteed or endorsed by the publisher.
