# Supplementary material for: Panobinostat Effectively Increases Histone Acetylation and Alters Chromatin Accessibility Landscape in Canine Embryonic Fibroblasts but Does Not Enhance Cellular Reprogramming
Source: Front Vet Sci. 2021 Sep 29;8:716570. doi: 10.3389/fvets.2021.716570 (PMC8511502; doi:10.3389/fvets.2021.716570)
Supplement: Supplementary file 1 [file Data_Sheet_1.docx]

Supplementary Material


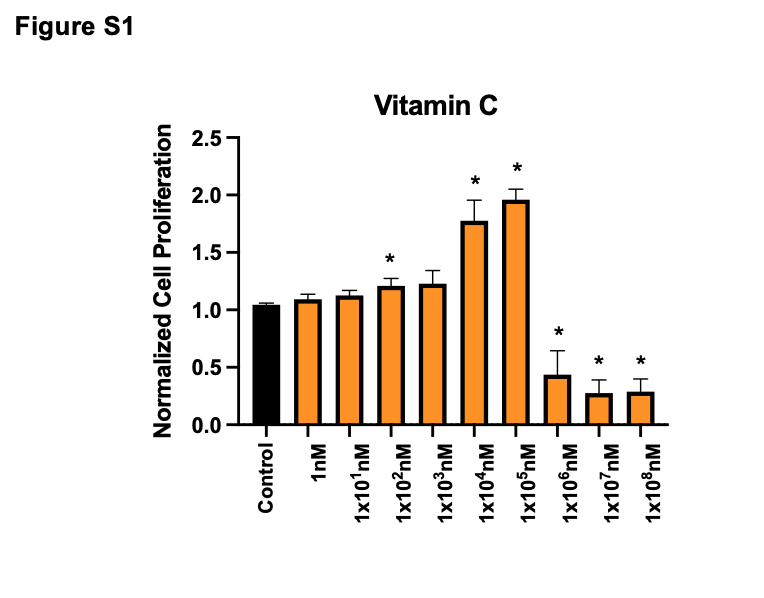


**Figure S1. Identification of non-cytotoxic concentration ranges of vitamin C in canine embryonic fibroblasts.**CEF were treated with a wide range of vitamin C concentrations for 48 hours. Cells were counted and data were normalized to the non-treated control. Each experiment was conducted in 3 CEF lines and was repeated twice. Concentrations higher than 100uM induced significant cytotoxicity. Data are presented as mean +/- SEM. The two-stage step-up method of Benjamini, Krieger and Yekutieli for controlling the False Discovery Rate(FDR) was used for multiple comparison correction. * FDR adjusted p-value < 0.05.


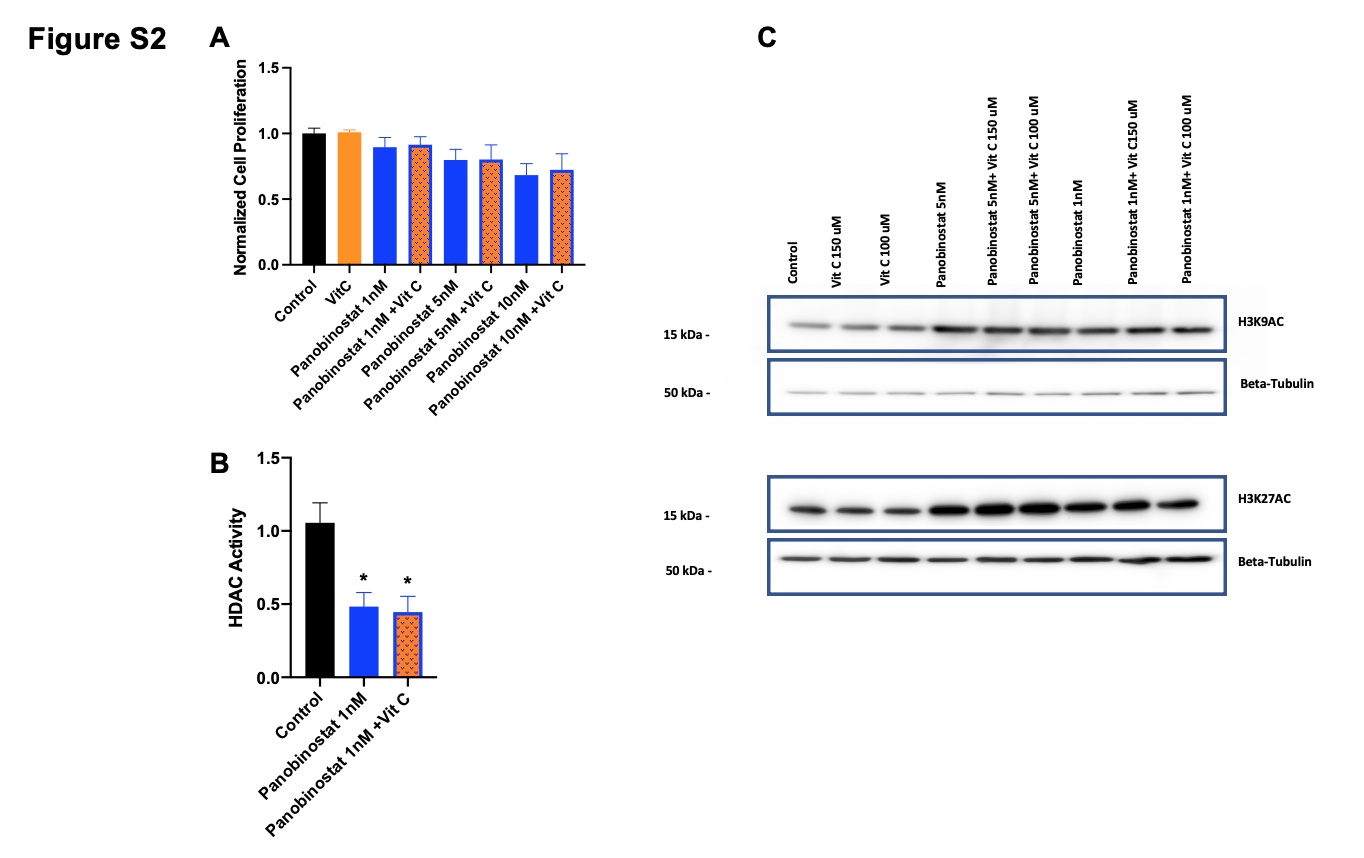


**Figure S2. The combination of vitamin C and 1nM panobinostat did not change the cell proliferation, HDAC activity, and histone acetylation.**CEF were treated with 150µM of vitamin C in combination with 1nM, 5nM, and 10nM panobinostat for 72 hours for MTS assay and 48 hours for HDAC activity and western blot for assessing histone acetylation. Data were normalized to the non-treated control. (A) Proliferation, the experiment was conducted in 3 CEF lines and was repeated twice. Data are presented as mean +/- SEM. There were no significant changes in the experiment. (B) HADC activity of the crude nuclear extract from 1x107 treated and non-treated cells was determined. Data are normalized to untreated control. The experiment was conducted in 2 CEF lines. Data are presented as mean +/- SEM. The two-stage step-up method of Benjamini, Krieger and Yekutieli for controlling the False Discovery Rate (FDR) was used for multiple comparison correction. * FDR adjusted p-value < 0.05.(C) Western blots showed no evident changes in histone acetylation of H3K9 (top) and H3K27(bottom) of CEF.All the protein samples were separated on one gel and the membrane cut based on the size of the β-tubulin (50 kDa) and either Acetyl-Histone H3 (Lys27) (15 kDa) or Acetyl-Histone H3 (Lys9) (15 kDa) for primary antibody incubation.

**
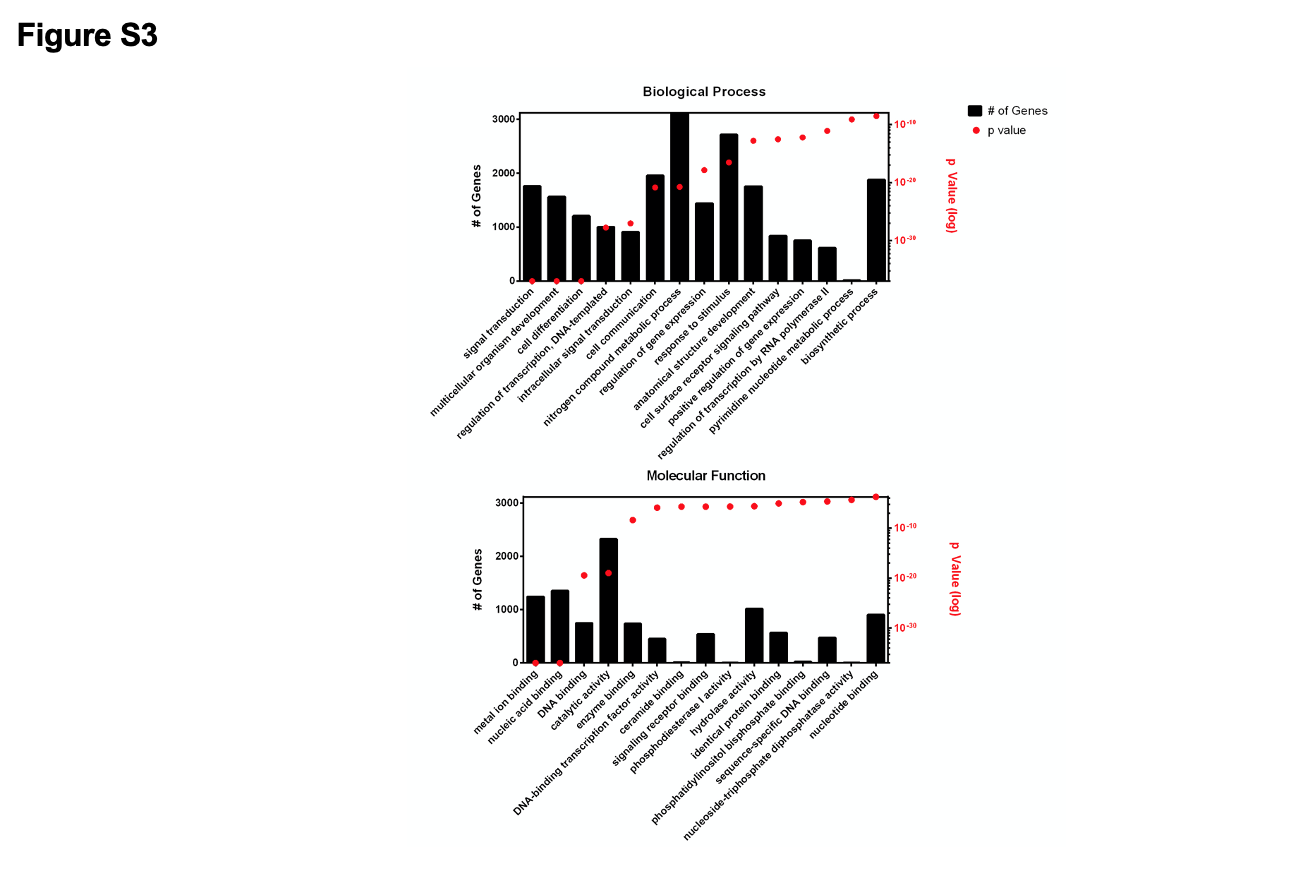
**

**Figure S3. GO Term categories and subcategories.** The first 15 GO terms with p<0.05 from each of “Biological Process” and “Molecular Function”, major ontologies, represent a view of the top GO terms enriched in the dataset.
